# The Role of Sodium-Glucose Co-Transporter-2 (SGLT-2) Inhibitors in Treating Lupus Nephritis: A Systematic Review

**DOI:** 10.7759/cureus.76130

**Published:** 2024-12-21

**Authors:** Stephanie Nagy, Marc M Kesselman

**Affiliations:** 1 Department of Rheumatology, Nova Southeastern University Dr. Kiran C. Patel College of Osteopathic Medicine, Fort Lauderdale, USA

**Keywords:** canagliflozin, dapagliflozin, empagliflozin, lupus membranous nephropathy, lupus nephritis, sglt-2 inhibitor, sodium-glucose transport protein-2, systemic lupus erythematosus

## Abstract

Systemic lupus erythematosus (SLE) is a prevalent autoimmune condition worldwide resulting from the loss of tolerance against self-antigens. The constitutional symptoms of SLE are well-known, including fatigue, fever, myalgia, weight loss, arthralgia, arthritis, malar rash, and photosensitivity. These symptoms often overshadow the impacts SLE can have on all body systems, with the renal system frequently impacted. Inflammation of the nephrons can progress to end-stage renal disease and renal failure if not treated effectively. Currently, the medications that are being utilized for the treatment of lupus nephritis (LN), include azathioprine, hydroxychloroquine, cyclophosphamide, belimumab, voclosporin, tacrolimus, mycophenylate, and rituximab. However, the majority of these medications are used off-label, and many come with severe side effects. With the additional benefits of anti-diabetic medications being examined outside of their original purpose, sodium-glucose co-transporter-2 (SGLT-2) inhibitors have begun to be investigated for their role in LN. To determine the impact of SGLT-2 inhibitors on LN risk and progression, a preliminary systematic review was conducted. A total of 248 articles were analyzed, with six being selected. SGLT-2 inhibitors were found to improve glomerular filtration rate (GFR), reduce proteinuria and albuminuria, and reduce the inflammatory cascade. The exploration of SGLT-2 inhibitors as a therapeutic strategy for LN represents a promising and innovative approach to managing this complex condition. SGLT-2 inhibitors show potential benefits beyond glycemic control. However, due to the novelty of this treatment and the limited studies completed, further testing is required to analyze its true effectiveness in LN.

## Introduction and background

Systemic lupus erythematosus (SLE) continues to be a prevalent rheumatological condition worldwide. SLE is an autoimmune multifactorial condition resulting from the loss of tolerance against self-antigens. Genetics, hormones, immune system abnormalities, and environmental factors all play a role in its development [1]. It is estimated that over 3.4 million individuals have been diagnosed with SLE, with a skewed prevalence seen in women. On average, the incidence per year is 5.41 per 100,000 (8.82 per 100,000 in women and 1.53 per 100,000 in men) [2].

As SLE’s etiology is multifaceted, it is difficult to pinpoint the exact trigger for each patient. As it is considered an autoimmune condition, defects in the immune system play a critical role in its development. Loss of tolerance against self-antigens leads to the development of autoantibodies, inducing inflammation throughout the body. Antinuclear antibodies (ANA) are most commonly found among SLE patients; however, they are also found in patients with other autoimmune conditions. As a result, if a patient is positive for ANA, more specific testing is completed for antibodies against double-stranded DNA (anti-dsDNA) or anti-Smith (anti-Sm) [3]. With the high inflammatory states, complement factors are consumed rapidly, resulting in low complements of C3 and C4 being noted in SLE patients [3]. Currently, numerous genes are being investigated for their involvement in the development of SLE; however, the major histocompatibility (MHC) locus that presents antigens, specifically HLA-DR2, HLA-DR3, HLA-DR15, has been found to have the greatest association thus far [4-7]. Hormones also play an impactful role, as women are at greater risk due to the impacts of estrogen. There is a significant increase in SLE in women with higher estrogen levels, early menarche, and who utilize estrogen-containing oral contraception or hormone replacement therapies [8-10]. This occurrence is said to be the result of estrogen stimulating the immune system by upregulating CD8 and CD4 T cells, B cells, macrophages, neutrophils, myeloperoxidase, lymphocytes, dendritic cells interleukins, MHC loci, and adhesion molecules of VCAM, ICAM, and PECAM [1,11]. The development of SLE is multifaceted, with the immune system, genetics, and sex all playing roles in its development; therefore, it is difficult to predict exactly who will develop SLE in the future as well as its progression in the body [3-11].

The impacts of SLE are not limited to a specific body system; however, the symptoms are widespread and present differently amongst patients, leading to misdiagnoses or late diagnosis. Constitutional symptoms in SLE patients include fatigue, fever, myalgia, and weight loss [12]. Arthralgia, arthritis, erythematous facial eruptions in a malar disruption frequently called a “butterfly rash”, and photosensitivity are the most commonly experienced symptoms of SLE. These symptoms often overshadow the impacts lupus can have on the cardiovascular, renal, gastrointestinal, and pulmonary systems as the condition progresses [12].

The renal impacts of SLE are the most prolific and present early in the disease progression. Lupus nephritis (LN) was found to occur in 38.3% of SLE patients. These patients had a higher incidence of death compared to patients with SLE who did not develop LN [13]. The incidence of LN is seen in certain sexes, ethnicities, and ages. Women have a higher prevalence of SLE; however, their male counterparts experience a higher risk of developing LN and more severe symptoms [14]. African Americans have a six- to seven-fold increase in developing LN as well as end-stage renal disease (ESRD) compared to Caucasians [15]. LN is additionally skewed to the younger population, as those diagnosed with SLE at a younger age, specifically children, have a greater risk for its renal complications [14,16]. Symptomology includes proteinuria, hematuria, oliguria, hypertension, edema, elevated creatine, and hypoalbuminemia [17].

LN is a type 3 hypersensitivity that occurs by immune complex (IC) formation, most commonly those composed of DNA and anti-dsDNA [17]. The deposition of the ICs within the mesangium, subendothelial, and subepithelial space of the kidney leads to an inflammatory reaction by the body [17]. The International Society of Nephrology/Renal Pathology Society classified LN into five progressive classes: Class I minimal mesangial involvement, Class II mesangial proliferation, Class III <50% involvement of the glomerulus, Class IV >50% involvement of the glomerulus, Class V membranous involvement and Class VI >90% involvement of the glomerulus [18]. As the severity of LN progresses, the treatments progress from observation to kidney transplantation. Those with Class I or II receive immunosuppressive therapy, and those with nephrotic range proteinuria will receive glucocorticoids and immunosuppressive therapy. Class III or IV receive immunosuppressive therapy of either mycophenolate mofetil (MMF) or cyclophosphamide or belimumab with either MMF or cyclophosphamide or calcineurin inhibitors like voclosporin with MMF. Class V or VI treatment dosages vary depending on the range of proteinuria, including renin-angiotensin system blockage, hydroxychloroquine, and immunosuppressive therapy with glucocorticoids and the addition of MMF or cyclophosphamide, calcineurin inhibitors, rituximab, and azathioprine in nephrotic range proteinuria [19,20]. However, glucocorticoids, voclosporin, and belimumab are the only FDA-approved medication for LN, all others are utilized off-label [21]. Despite the pharmacological development, 10-30% of LN patients will progress to ESRD, requiring transplantation [22]. Due to this, additional therapeutic managements are sought to improve the outcomes for these patients.

Sodium-glucose co-transporter-2 (SGLT-2) inhibitors traditionally have been utilized as antidiabetic medications. SGLT-2s are in charge of actively transporting glucose across the luminal membrane into the intracellular fluid in exchange for sodium. By inhibiting this transport protein, glucose is restricted from being reabsorbed into the body, resulting in its excretion in the urine [23]. This class of antidiabetics has recently been found to have a nephroprotective factor. Inhibition of glucose reabsorption allows sodium to pass through the nephron, triggering the macula cells to release adenosine, which vasoconstricts the afferent glomeruli, reducing intraglomerular pressure [24].

With the limited effectiveness of current LN treatment guidelines and the nephroprotective factors of SLGT-2 inhibitors, this review aims to analyze the current literature examining the impact that SGLT-2 inhibitors may have on the progression and outcomes of LN.

## Review

Methods

Search Strategy

A systematic literature review was performed using the Cumulative Index to Nursing and Allied Health Literature (CINAHL), Ovid, Excerpta Medica Database (EMBASE), Web of Science, and Google Scholar using the search term “Sodium-glucose cotransporter-2 inhibitors” OR “SGLT-2 inhibitors” AND “lupus nephritis.” To ensure the relevancy of the articles, those published between 2010 and 2024 were assessed. The articles were analyzed in a step-wise process, first evaluating the title and abstract, then further assessing the full-text manuscript to ensure the articles analyzed patients diagnosed solely with LN. Nova Southeastern University’s library database was used to access databases and full-text articles.

Selection Criteria

**Figure 1:**
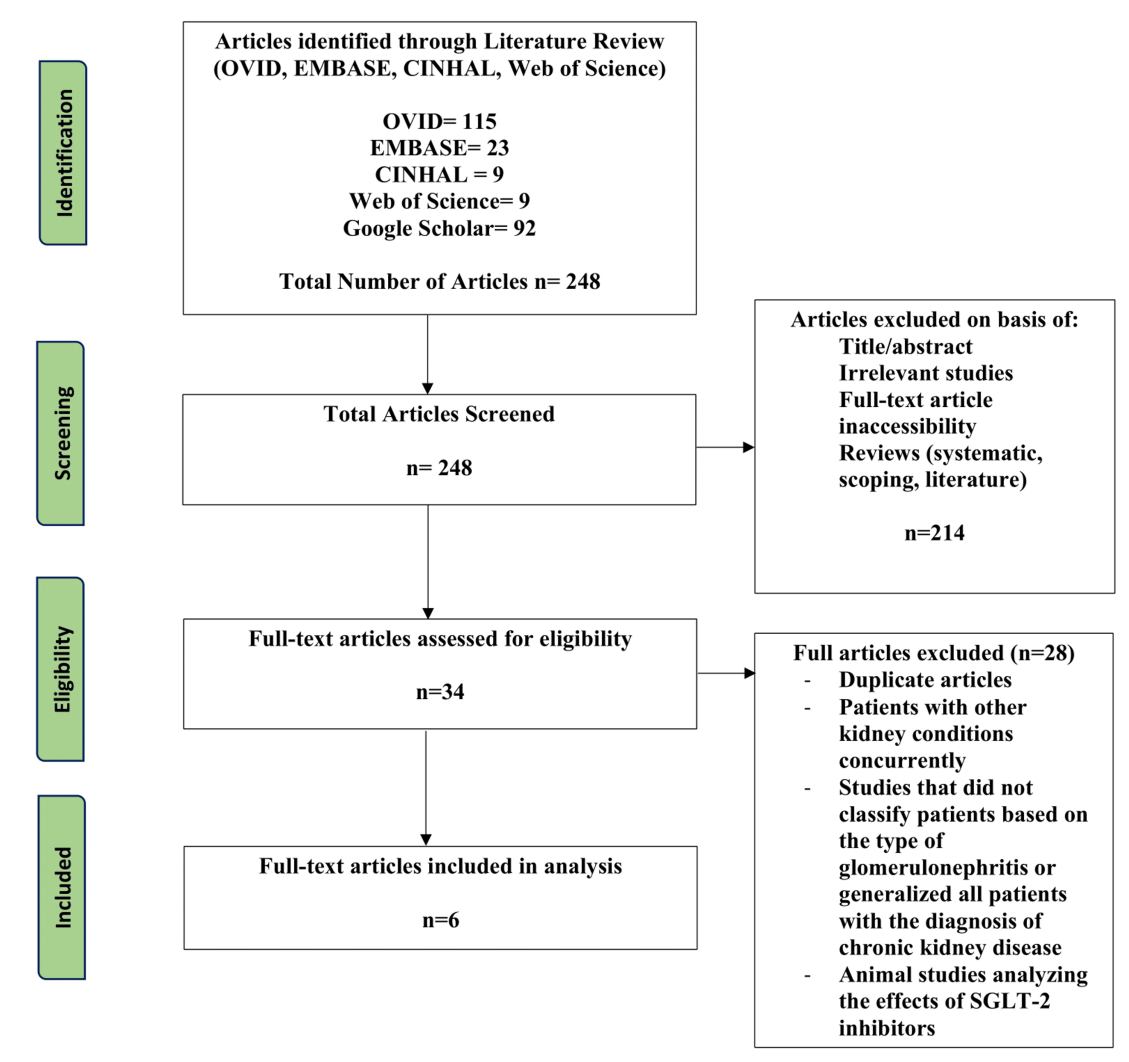
PRISMA diagram for selection criteria PRISMA: Preferred Reporting Items For Systematic Reviews and Meta-Analyses; CINAHL: Cumulative Index to Nursing and Allied Health Literature; EMBASE: Excerpta Medica Database; SGLT-2: sodium-glucose co-transporter-2

The study designs included randomized control trials, cross-sectional studies, observational studies, and cohort prospective/retrospective studies. Exclusion criteria included study designs of literature, systematic or scoping reviews, and animal studies. Articles were removed if the patients had additional renal diagnoses other than LN, and if articles did not specify the type of glomerulonephritis or if all patients were grouped with the diagnosis of chronic kidney disease but failed to clarify the initial diagnoses that led to the renal failure. The articles were assessed using the Joanna Briggs Institute critical appraisal checklists [25]. The Preferred Reporting Items for Systematic Reviews and Meta-Analyses (PRISMA) were used to develop a flow diagram of the selection criteria for reproducibility (Figure 1) [26].

Results

In total, 248 articles were populated between the databases of CINHAL, OVID, EMBASE, Web of Science, and Google Scholar. After the first-tier screening, 214 articles were removed based on title, abstract, full-text availability, study type, publication year, and English language availability. Thirty-four articles were eligible for the second round of screening, in which full texts were completely screened. Due to the novelty and recency of this field, 12 were found to be duplicates, and two studied mice. The others examined the impact of SGLT-2 inhibitors in glomerulonephritis or chronic kidney disease without specifying the exact diagnoses of the patients and due to the unknowns within these articles they were removed as well, leaving only six to be analyzed. With the originality of SGLT-2 inhibitors used as a treatment method for LN, it was expected the articles gathered would be limited; however, it is crucial to present this preliminary review to make clinicians aware of the further nephroprotective features of this class of medication.

**Figure 2:**
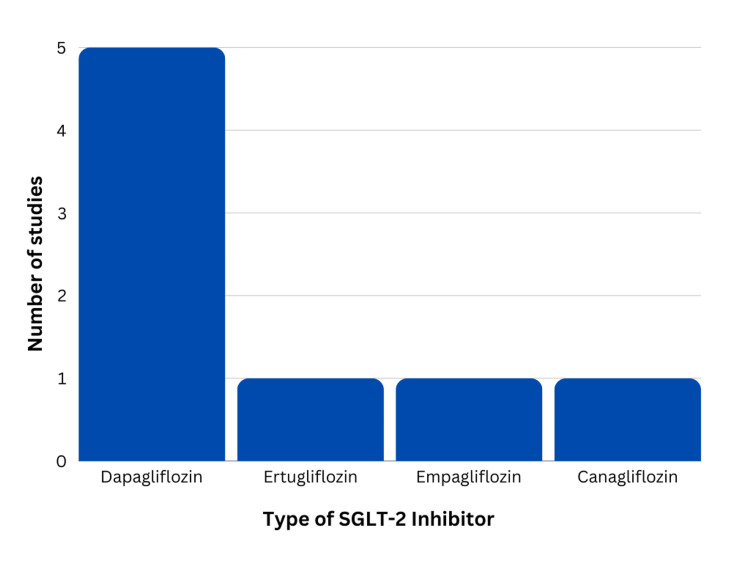
SGLT-2 inhibitor medications used within the studies SGLT-2: sodium-glucose co-transporter-2

**Figure 3:**
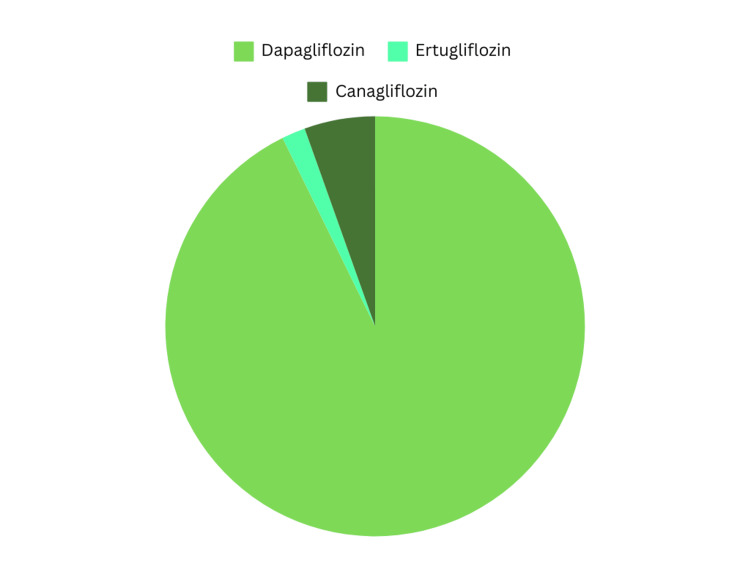
Analyzing the reported number of patients receiving each SGLT-2 inhibitor SGLT-2: sodium-glucose co-transporter-2

Table 1 depicts the studies analyzed, including the number of patients, age, specific SGLT-2 inhibitor received, and the findings of the articles. In total, 3648 patients were analyzed with 1831 being diagnosed with LN. In total, the majority were females (3090, 84.7%), while males were 558 (15.3%). The ages of the patients ranged from 34.51 to 56.8, with a mean age of 42.1. Almost all articles reported the type of SGLT-2 inhibitor received, except for Yen et al. [27]. In the articles that were reported, patients received dapagliflozin, canagliflozin, ertugliflozin, and empagliflozin. Figure 2 indicates the usage of each SGLT-2 inhibitor with dapagliflozin at 10 mg being the most commonly utilized among patients with LN [28-32]. Out of the six articles, five reported the type of SGLT-2 inhibitor used; however, only four specified how many patients received each one. Elkeraie et al. analyzed patients who received dapagliflozin 10 mg or empagliflozin 10-25 mg but, failed to indicate the number of patients in each group, and Yen et al. did not specify the type of SGLT-2 inhibitors used [27,32]. In the remaining studies that clearly indicated the treatments received by patients, 51 received dapagliflozin 10mg (92.7%), three received 100 mg of canagliflozin (5.5%), and one received 5 mg of ertugliflozin (1.8%). Figure 3 highlights the increased usage of dapagliflozin amongst patients compared to other SGLT-2 inhibitors. Even with the exclusion of Yen et al. and Elkeraie et al., due to failure to specify, it is clear that dapagliflozin by far is the most commonly prescribed medication among patients with LN [27,32].

**Table 1:** Characteristics of the studies examined SGLT-2: sodium-glucose co-transporter-2; GFR: glomerular filtration rate; eGFR: estimated glomerular filtration rate

Article	Year	Number of Patients	Average Age	Treatment Received	Findings
Yen et al. [27]	2024	3550 (1775 control 1775 SGLT-2 inhibitor); SGLT-2 users (1514 F, 225M, 36 unknown)	56.8	Not specified	Significantly lower risk of LN (p<0.001) (CI 0.40-0.77)
Zhao et al. [29]	2023	9 (6 F, 3 M)	37	Dapagliflozin 10 mg (n=5); canagliflozin 100 mg (n=3); ertugliflozin 5 mg (n=1)	Significant reduce proteinuria; Increase in GFR levels; Increase in serum albumin.
Wang et al. [28]	2022	38 (36 F, 2 M) (17 with lupus nephritis (LN), 21 without LN)	34.51	Dapagliflozin 10 mg for six months	No reduction in proteinuria (1.7g at baseline and after treatment) in LN patients; Significant elevation of 8.26 g/L in hemoglobin for LN patients; Significant loss of BMI in LN patients from 23.78kg/m^2^ to 23.28kg/m^2^; Significant reduction in systolic blood pressure from 121. 32 to 115.97 mmHg; Improved eGFR in patients who have a GFR <0.90 mL/min/1.73m^2^
Vajgel et al. [30]	2023	20 (18 F, 2 M)	39.5	Dapagliflozin 10 mg for 24 weeks	Non-significant reduction of proteinuria at three months and six months
Vajgel et al. [31]	2024	14 (12 F, 2 M) (nine in treatment, five in standard care)	38.3	Dapagliflozin 10 mg	Reduced gene expression of IL-1 (p=0.017), but no change in NLRP3 (p=0.73) expression after six months of dapagliflozin
Elkeraie et al. [32]	2023	17 (6 F, 11M); Patient with glomerulonephritis but only one with LN	46.7	Dapagliflozin 10 mg or empagliflozin 10-25 mg	Serum creatinine significantly decreased from 1.7 to 1.37 mg/dL; eGFR significantly improved from 59 to 62 mL/min/1.73m^2^; Albumin to creatine ratio decreased from 2669 to 858 mg/g; Albumin level stayed constant at 3.7 g/dL

Yen et al. analyzed the impact of SGLT-2 inhibitors on developing certain conditions within patients with SLE, and regarding LN, they found a significant reduction in the risk of its development (p<0.001) [27]. A reduction in proteinuria was noted by Zhao et al., but Wang et al. and Vajgel et al. reported no significant decrease [28-30]. An improvement in glomerular filtration rate (GFR) was seen in all studies that analyzed its outcomes [28,29,32]. Changes in albumin levels varied with Zhao et al., finding an elevation, while Elkeraie et al. noted consistent levels, however, with reduced albumin-to-creatinine ratio [29,32]. Vajgel et al. analyzed the immune system markers that are impacted by SGLT-2 inhibitors and found a significant reduction in IL-1 (p=0.017), a major inflammatory cytokine, but no change in NLRP3 inflammasome responsible for the secretion of IL-1 [31].

Discussions

LN continues to be one of the most detrimental outcomes of SLE, with more than 30% of patients developing it after three years of initial diagnosis. Despite an increase in survival rates amongst this patient group, more than 20-32% never obtained remission, resulting in a 10-fold increase in ESRD and chronic kidney disease. Among those that reached remission, 32-55% developed a lupus flare in the years following [33-35]. As a result, it is crucial to adapt current regimens to find effective treatments that limit the risks of LN development and life-threatening progression to renal failure [13].

The kidneys play a critical role in the filtration of fluids to excrete toxins, waste products, and excess ions. IC deposition is the most common cause of LN, with a lesser-known source occurring in the absence of IC by inflammatory cytokines. Depending on the depth of penetration of the IC, the severity of LN can vary. Deposition into the subendothelium with access to the vasculature results in classes III and IV, while subepithelial deposits damage the podocytes and result in class V. Rupturing of the glomerular basement membrane can introduce IC into the glomerulus, inducing the complement cascade [36]. After IC deposition, inflammatory cytokines are summoned to the site to induce an inflammatory cascade of type 1 IFN, IFN-gamma, TNF-alpha, nitric oxide, IL-1, and IL-17, causing vascular refraction and podocyte effacement and loss [36]. Due to the inflammatory nature of this disease, treatment has often targeted the immune system.

Conventionally, high-dose corticosteroids with either MMF or cyclophosphamide are used in the treatment of LN; however, only corticosteroids and recently voclosporin and belimumab are FDA-approved for LN. Corticosteroids rapidly reduce inflammation but are associated with their fair share of side effects, including weight gain, hirsutism, edema, hypertension, blurry vision, and depression [21]. There has also been a push to transition to lower doses of steroids and/or quicker tapering of steroids to lessen the side effects experienced [37]. The 2019 European Alliance of Associations for Rheumatology (EULAR) and European Renal Association-European Dialysis and Transplant Association (ERA-EDTA) guidelines, along with the 2021 Kidney Disease: Improving Global Outcomes (KDIGO) guidelines recommend the utilization of lower dosages or steroids followed by a secondary therapy to facilitate faster withdrawal from corticosteroids [37]. Cyclophosphamide, when paired with corticosteroids, reduces kidney failure risk; however, it has a significant risk of ovarian failure and malignancies. As a result, the search continued for safer alternatives [21]. MMF was found to be less toxic and not pose the same malignancy risk as cyclophosphamide and has recently been replaced in LN treatment, but it poses gastrointestinal risks [21]. Even hydroxychloroquine, an antimalarial, has been trialed due to its effect on blocking the signaling of dendritic cells, type 1 IFN, and proinflammatory cytokines. Hydroxychloroquine has reduced LN flare-ups and delayed its progression; however, due to the severe risk factor of macular damage, this too is not a suitable option for all [21].

Voclosporin and belimumab are the only drugs approved by the FDA for the treatment of LN. A new drug that is undergoing investigational therapy is ATA3219, an allogenic CD19-targeting chimeric antigen receptor T cell (CAR-T) therapy, it recently received FDA approval to conduct a phase 1 clinical trial on LN following their previous trial on SLE. Current medications frequently used in the treatment of LN off-label include MMF, rituximab, tacrolimus, corticosteroids, azathioprine, methotrexate, hydroxychloroquine, and cyclophosphamide. Most recently, voclosporin was the first oral therapy approved for LN due to it meeting the renal endpoints earlier of a protein-to-creatinine ratio of less than 0.5, GFR greater than 60 mL/min/1.73m^2^ and meeting the limitations of prednisone use, also it rapidly reduced proteinuria with only a mild reduction in GFR rates [37-39]. Belimumab is the only IV medication for LN that met renal endpoints of a protein-to-creatinine ratio of less than 0.7, GFR greater than 60 mL/min/1.73m^2^, and had a 50% reduction in renal-related events of deaths [37,40]. Progress has recently been made in finding more suitable medications; however, the search continues to find effective and safe alternative medications that can reduce the risk of LN as well as halt its progression to ESRD. Anti-diabetic drugs have recently been at the forefront of investigating their alternative medical benefits, including SGLT-2 inhibitors.

Traditionally, the nephroprotective features of other antidiabetic medications are unmatched when compared to SGLT-2 inhibitors. Insulin was found to reduce creatinine levels but had no impact on the risk reduction of ESRD. Metformin has been found to reduce reactive oxygen species and, thus, hypoxic damage of nephrons. Sulfonylureas and DDP-4 had no renal protective features. Glucagon-like peptide-1 receptor agonists (GLP-1 RAs) indicated some nephroprotective features by reducing levels of macroalbuminuria and creatinine levels; however, they had no impact on GFR. However, the limiting of the GFR decline and progression to ESRD was only seen amongst SGLT-2 inhibitors [41,42].

SGLT-2 inhibitors are thought to work on the renal system in numerous ways, attributed to their protective nature. The reduction in intraglomerular pressure by constricting the afferent glomeruli is believed to be their most protective aspect [24]. SGLT-2s are located on the proximal convoluted tubule in the nephron and reabsorbed almost 90% of the glucose filtered in the nephron by actively moving sodium out of the cells and into the nephron. Inhibiting this transporter leads to glucosuria and improves hyperfiltration, which reduces intraglomerular pressure [41]. With glucosuria, serum glucose levels decrease, reducing advanced glycation end products, thus lessening oxidative stress and inflammation [43]. SGLT-2 inhibitors reduce blood pressure by eliminating excessive fluid through its natriuresis and glucosuria which further protects the kidneys, as seen in Wang et al. [28].

Most significantly, the elevated intraglomerular pressure with LN over time leads to glomerular hypertrophy, glomerulosclerosis, and nephron loss, leading to albuminuria and proteinuria, which increases tubulointerstitial inflammation and fibrosis [44]. Proteinuria must be present to clinically diagnose LN. More than 50% of LN patients have nephrotic range proteinuria, >3.5 grams; however, levels above 500 mg already contribute to kidney inflammation [20,21,45]. Studies have further shown that groups with higher severity of LN displayed greater levels of albuminuria [46]. With SGLT-2 inhibitors, Zhao et al. noted greater serum albumin levels, indicating a reduction in the amount of albumin excreted, while Elkeraie et al. saw a consistent level of albumin [29,32]. In regard to proteinuria, Zhao et al. found a significant reduction in proteinuria, while Vajgel et al. and Wang et al. noted consistent levels of proteinuria [28-30]. Thus, with the limited research done on SGLT-2 inhibitors, further research is required to fully and completely analyze their true effectiveness on proteinuria and albuminuria.

Customarily, kidney function is analyzed by GFR and creatinine clearance. Even with the current standard of care, 30% of LN patients experience continued GFR decline [47]. By the time GFR falls below 75 mL/min/1.73m^2^, it is the greatest risk for the progression of LN to chronic kidney disease. Current research has found that SGLT-2 inhibitors were found to significantly improve GFR across all studies [28,29,32]. Patients with LN showed significant serum creatinine levels and protein-to-creatinine ratios which are associated with a declining state in LN. SGLT-2 inhibitors have been shown to reduce serum creatinine and the albumin-to-creatine ratio, thus indicating improved kidney function [28,32,48]. It is promising that SGLT-2 inhibitors could significantly improve kidney function by elevating GFR and reducing serum creatine.

SGLT-2 inhibitors were associated with not only improving renal outcomes but also with improving the kidneys on a cellular level. Podocytes are the epithelial cells of the Bowman’s capsule that assist in blood filtration; however, they are very susceptible to damage. Proliferative LN targets the podocytes, resulting in actin cytoskeleton dysfunction, loss of podocyte-specific markers, cell detachment, and mitotic failure. This loss can also trigger the immune system through T-cell activation and NLRP3 inflammasome activation, triggering the release of IL-1 [49]. The NLRP3 pathway plays a significant role in the inflammatory response within LN, releasing inflammatory cytokines of IL-1 and caspase-1. Elevated levels of NLRP3 were associated with greater podocyte injury and high rates of proteinuria [50]. SGLT-2 inhibitors have been found to reduce the activation of mTOR, thus reducing the activation of NLRP3-mediated inflammation, which decreases podocyte injury [29]. Also, Vajgel et al. found a significant reduction in the inflammatory cytokine of IL-1; however, the NLRP3 levels remained constant after six months of dapagliflozin [31]. Due to the limited research in this area, more studies are required to see the impact of SGLT-2 inhibitors on podocytes and the inflammatory cascade but early research indicates its initial benefits.

The limitations of this review are the small number of studies analyzed due to the novelty of this area of research, leading to the inability to fully analyze the impact of SGLT-2 inhibitors on LN. Additionally, the small sample size used within the articles also limits its generalizability to the large population of SLE patients who develop LN. Future research should focus on large-scale, randomized controlled trials to validate the benefits of SGLT-2 inhibitors in LN. These studies should aim to clarify the mechanisms underlying their renal protective nature and evaluate their impact on long-term clinical outcomes. Finally, as patients with SLE are susceptible to infections, they may be at a higher risk of developing urinary tract infections (UTIs) which are common side effects of SGLT-2 inhibitors. Only one study analyzed the side effects within patients noting that there was no elevated risk of developing UTIs with this medication [28]. Further research is required to investigate any additional side effects that may be experienced.

The use of SGLT-2 inhibitors as treatment for LN is in its early stages; however, this preliminary review was critical to illuminate the initial research conducted and its promising ability to improve kidney function in this vulnerable group. Further research is required to truly understand the additional benefits outside of its traditional use as an anti-diabetic medication as well as side effects in immuno-compromised patients.

## Conclusions

The exploration of SGLT-2 inhibitors as a therapeutic strategy for LN represents a promising and innovative approach to managing this complex condition. SGLT-2 inhibitors show potential benefits beyond glycemic control. Evidence suggests that these agents may positively influence renal outcomes by reducing glomerular hyperfiltration and intraglomerular pressure, improving GFR, decreasing proteinuria, and exerting anti-inflammatory effects. The use of SGLT-2 inhibitors in LN holds substantial promise. Continued research and clinical trials will be pivotal in determining their definitive role in managing LN and potentially improving patient outcomes. Isolating biomarkers that will help determine which patients may benefit the most from SGLT-2 inhibitors is of utmost importance going forward. Making SGLT-2 inhibitor therapy a standard for all patients with SLE is another preventive consideration, and more research will be needed to validate this.
